# The Effect of Health Check-Ups on Health Among the Elderly in China: Evidence From 2011–2018 Longitudinal Data

**DOI:** 10.3389/ijph.2022.1604597

**Published:** 2022-08-05

**Authors:** Dantong Zhao, Zhongliang Zhou, Chi Shen, Xiaohui Zhai, Yaxin Zhao, Dan Cao, Qiwei Deng, Guanping Liu, Jeremy Fung Yen Lim

**Affiliations:** ^1^ School of Public Policy and Administration, Xi’an Jiaotong University, Xi’an, China; ^2^ School of Public Health, Health Science Center, Xi’an Jiaotong University, Xi’an, China; ^3^ Saw Swee Hock School of Public Health, Leadership Institute for Global Health Transformation, National University of Singapore, Singapore, Singapore

**Keywords:** health, elderly, urban-rural difference, health check-ups, preventive care

## Abstract

**Objectives:** To assess the effect of health check-ups on health among the elderly Chinese.

**Methods:** The first dataset was panel data extracted from the 2011, 2014, and 2018 waves of the Chinese Longitudinal Health Longevity Survey (CLHLS). The second dataset was cross-sectional data come from CLHLS 2018 linked with the lagged term of health check-ups in CLHLS 2011. Health check-ups were measured by a binary variable annual health check-up (AHC). Health was assessed by a binary variable self-rated health (SRH). A coarsened exact matching method and individual fixed-effects models, as well as logistic regressions were employed.

**Results:** AHC attendance among the elderly increased from 2011 to 2018, with higher utilization of AHC also detected in the rural group. AHC had positive effects on SRH among rural respondents (short-term effect: OR = 1.567, *p* < 0.05; long-term effect: OR = 3.385, *p* < 0.001).

**Conclusion:** This study highlights a higher utilization of AHC in rural area, and the effectiveness of AHC in SRH improvement among rural participants. It indicates enhanced access to public healthcare services in rural area and underlying implications of health check-ups for reducing urban–rural health inequalities.

## Introduction

“Prevention first” has been one of the major healthcare policies in China since the 1950s and is considered to have been successful in improving the health status of China’s population [1]. Utilization of preventive care has contributed to both reducing premature mortality and improving quality of life [2]. In China, the national basic public health service program (NBPHS) is regarded as a long-term preventative health policy. Funded by the government, NBPHS packages (e.g., infant care, maternal care, elderly care) are provided free of charge to all residents, regardless of location [3]. The NBPHS policies not only cut medical cost but also increase basic public health services (BPHS) coverage and reduce disparities between areas of higher and lower economic development, improving the health status of both urban and rural residents [4].

Under the NBPHS elderly care package, all residents aged ≥65 years are eligible for free annual health check-up (AHC) [5]. Height, weight, blood pressure (BP), blood and urine routine, lipids, glucose are measured during health check-ups. Then, based on the results, participants receive related health education and can ask for health counseling. Similar to China, Japanese adults aged ≥40 years are provided with a free AHC covering similar examination elements [6]. Moreover, the U.S. Preventive Services Task Force (USPSTF) recommends regular monitoring for adults with high BP, lipid disorders, and obesity [7]. There is evidence to suggest that regular check-ups are conducive to early detection and decrease of risk factors (e.g., total cholesterol, body mass index (BMI), BP and uric acid [8–10]), as well as prolonged life-span in many developed countries [11].

It is a fact that age brings greater likelihood of health problems. Thus, the health of elderly individuals is more vulnerable. Health check-ups are regarded as a key strategy in the framework of healthy and active ageing. Several studies in Western countries have concluded that regular health check-up attendance by the elderly led to a general improvement in their health, resulting in reduced demand for curative care [12], and an increase in outpatient medical expenditures but a decrease in inpatient medical expenditures [13]. In Asia, a study of elderly Taiwanese found a negative relationship between regular health examination and hospitalization care regarding the length of stay and medical expenditures [14]. Another study in Taiwan also indicated that utilization of preventive care services was conductive to reducing the need for inpatient services by the elderly [15]. However, any relationship between regular health check-ups and health among the elderly on mainland China is largely unknown. Thus, this study concentrates on old Chinese adults. We expect to find that health check-ups have positive effect on health.

On the background of the dual Chinese urban-rural social structure, rural and urban populations are economically and socially given different treatments. This leads to socioeconomic disparities between rural and urban residents. Rural residents in China are more likely to be farmers with lower educational attainment and income [16], and also less access to government-sponsored public resources or healthcare services [17], compared to their urban counterparts. This is especially true among the elderly. Under the NBPHS, all older adults, regardless of the rural or urban residence restriction, are eligible for a free AHC, in a bid to reduce the urban–rural gaps in access to health services and to equalize BPHS. Preventive care has the potential to progressively benefit the poor [18]. Those rural residents with lower socioeconomic status may feel more inclined to utilize health services than they would have been the previous billed services. Thus, we expect the effect of health check-ups on health may differ between rural and urban old adults, and that rural participants may benefit significantly.

Given the lack of discussion about the effect of health check-ups on health among the elderly in China, and the disparities between rural and urban areas, we are attempting to contribute to the literature on health check-ups and health among older adults in China from an urban-rural perspective. This study aims to explore the magnitude and trend of AHC utilization over time, to assess the effect of AHC on health among the elderly, and to examine the urban-rural differences.

## Methods

### Study Sample

**FIGURE 1:**
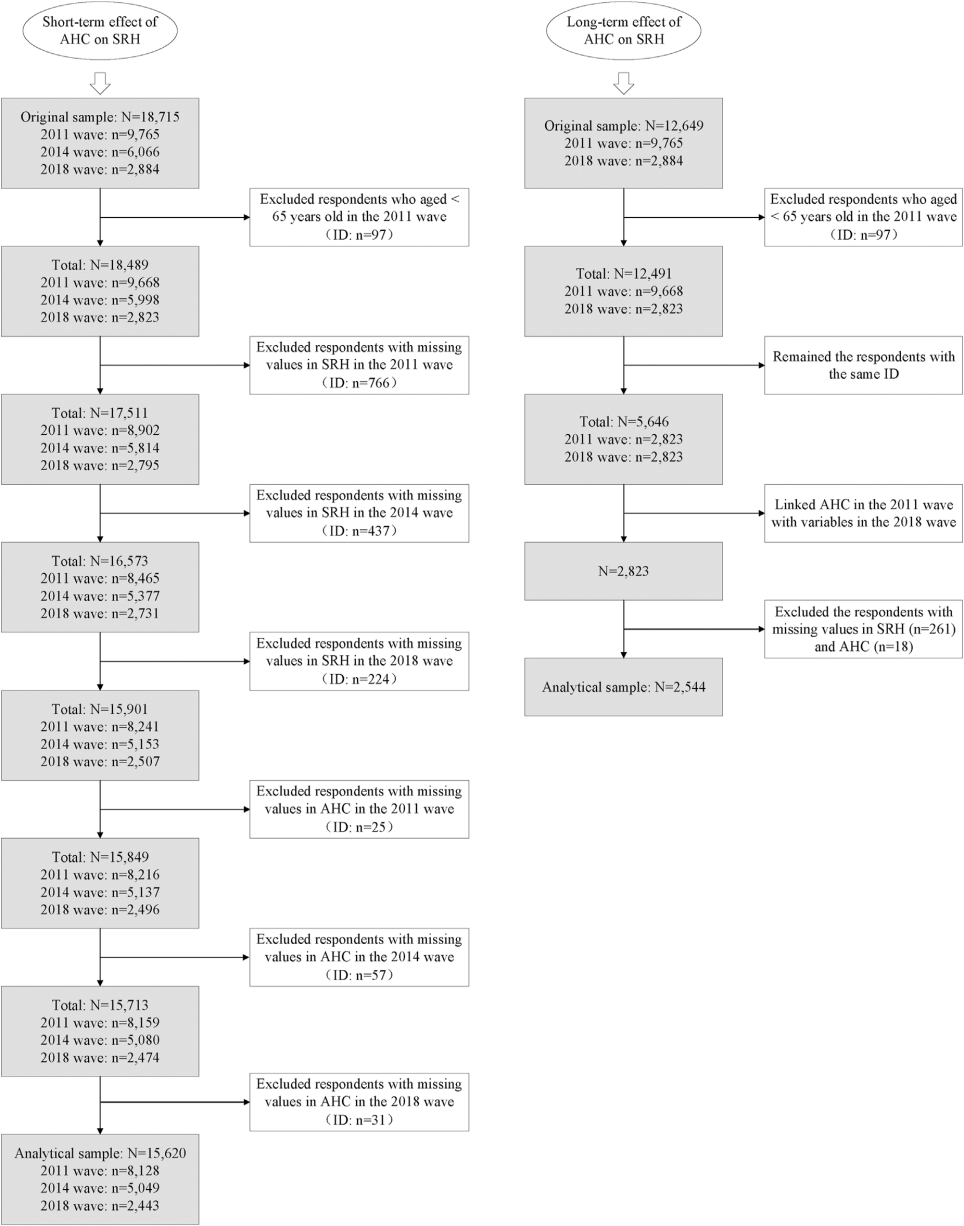
Flow chart of analytical sample selection procedure (Chinese Longitudinal Health Longevity Survey, China, 2011, 2014, 2018).

The longitudinal data used in this study came from Chinese Longitudinal Healthy Longevity Survey (CLHLS), a dynamic, nationally representative cohort survey that investigates the determinants of health and longevity of elderly Chinese. The baseline survey of the CLHLS was conducted in 1998, followed by seven survey waves—in 2000, 2002, 2005, 2008, 2011, 2014 and 2018—covering 23 of the 31 provincial administrative units. It constituted 85% of the total population and covered the eastern, middle, western and northern regions, as well as the northeast China [19]. Zeng et al. provided a detailed look at the CLHLS, including sampling methods, procedures, follow-up interviews, and data quality [20]. Two datasets were used in this study. The first was the three most recent waves of data because AHC, a key explanatory variable for the current study, did not start being carried out until 2009. The longitudinal survey covered 9,765 respondents in 2011. In follow-up surveys in 2014 and 2018, respectively, 6,066 and 2,884 respondents were re-interviewed. We excluded all individuals aged <65 years and all those who failed to respond to the key explanatory and explained variables. This resulted in a non-balanced panel of 15,620 individuals aged ≥65 years to use for final analysis. The number of respondents remaining in follow-up surveys was 8,128 in 2011, 5,049 in 2014, and 2,443 in 2018. The sample split between rural and urban participants was 7,949 and 7,671, respectively. The second dataset was collected from CLHLS 2018, which was matched with a lagged term of health check-ups in CLHLS 2011. There were 2,544 respondents entered final analytical sample, with 1,222 rural participants and 1,322 urban participants. Analytical sample selection procedure is outlined in Figure 1.

### Measurements

In this study, self-rated health (SRH) served as a dependent variable, which has proven to be a good indicator of objective health as well as a sensitive and reliable predictor of health-related behaviors and health-care demand, particularly for the elderly [21]. SRH was drawn directly from the question, “How do you rate your health at present?” Possible answers were “very bad,” “bad,” “fair,” “good” and “very good”. We dichotomized the variable into “Good” health (including “fair”, “good” and “very good”) and “Poor” health (including “bad” and “very bad”) in accordance with previous studies, coding them 1 and 0, respectively [22, 23]. The key independent variable was AHC, measured by question, “Do you have a regular health check-up once every year?” If the respondents answered “Yes,” we then coded 1; otherwise, we coded 0.

As a key variable, rural or urban residency was defined in this study by question, “What is your current residence area?” Urban residents were persons who resided in cities or towns, and rural residents were those who lived in rural areas [24]. Control variables included age (65–79 years old, ≥80 years old), gender (male, female), marital status (separated or divorced or widowed or never married, married and living with spouse), education level (illiterate, literate), pre-retirement occupation (white collar, others), region (eastern, central south, west, north, northeast), economic status (poor, median, richer), suffering from activities of daily living (ADLs) disability (yes, no), suffering from instrumental activities of daily living (IADLs) disability (yes, no), social security and insurance (yes, no), number of chronic diseases (none, one, two or more), access to healthcare services (yes, no), cohabiting with others (yes, no), smoking (yes, no), drinking (yes, no), exercising (yes, no). In line with World Health Organization (WHO), as well as previous studies [25–27] and a sample distribution, 80 was used as the cut-off age for dividing group participants, to distinguish the “young-old” from the “oldest-old”. Education level was measured by years of schooling. Those individuals with no schooling were defined as illiterate, while everyone else was literate [28]. Whether or not they had been a white-collar worker was drawn from the question, “What was your primary occupation before age 60?” We dichotomized the variable into white collar (professional or technical, governmental or institutional or managerial, industrial, and military personnel) and others (self-employed, agricultural, unemployed personnel, and house workers) [29]. The geographic regions included in the CLHLS were categorized into the following parts: east (Jiangsu, Zhejiang, Anhui, Shanghai, Fujian, Jiangxi, and Shandong); central-south (Hainan, Guangxi, Guangdong, Henan, Hunan, and Hubei); west (Shaanxi, Sichuan, and Chongqing); north (Hebei, Shanxi, Beijing, and Tianjin); northeast (Heilongjiang, Liaoning, and Jilin) [30]. Economic status was collected using the question, “How do you rate your economic status compared with others in your local area?” on a five-point scale (“very rich,” “rich,” “fair,” “poor,” “very poor”). On account of the small variation that we found in this variable, we combined “rich” and “very rich” into “rich,” and “poor” and “very poor” into “poor,” to give economic status three categories: “Rich,” “Median,” and “Poor.” ADLs were assessed according to whether the respondent could independently perform dressing, bathing or showering, eating, getting into or out of bed, using the toilet, and controlling urination and defecation. IADLs were evaluated by whether the respondent encountered difficulties with doing chores, preparing hot meals, shopping for groceries, managing personal finances, making phone calls, and taking medications. Individuals were defined as having a disability when they had trouble doing any of those tasks. Chronic diseases were measured by question, “Are you suffering from any of the following 24 chronic diseases?” We calculated the number of diagnosed chronic diseases, and classified it into “none,” “one” and “two or more.” Access to healthcare services was assessed with the question, “Can you get adequate medical service when you are sick,” with an answer of “Yes” or “No.”

### Statistical Analysis

To fully understand the role of health check-ups on health, we examined the short- and long-term effects of AHC on SRH. In the process of short-term effect detection, we first used the full panel sample to get an idea of the crude association between AHC and SRH. We then extracted anyone who did not participate in AHC in the baseline 2011 wave. They were classified into two groups—those who attended AHC and those who did not—according to whether they underwent AHC in any follow-up year. A coarsened exact matching (CEM) method was employed to preprocess the data in order to ensure that individuals who underwent AHC had the “same” health conditions and socioeconomic characteristics as those who did not in the baseline year (2011), eliminating the influence of health condition and socioeconomic characteristics on AHC utilization [1, 31, 32]. As there are no applicable policy changes or other forms of natural experiments, quasi-experimental matching methods become a viable strategy for drawing causal inferences [33]. It has been demonstrated that CEM dominates other matching methods (e.g., propensity score matching (PSM)) in its ability to increase efficiency and reduce imbalance, model dependence, estimation error, variance, and bias [21]. CEM ensures that any imbalance between the matched treated and control groups is not larger than the *ex ante* user choice, and that improvements in the bound on balance for one covariate will not affect the maximum imbalance of each of the other covariates [34, 35]. The user does not need to conduct any further balance checking or restrict data to common support, as is required by PSM [35, 36]. In the present study, CEM was employed based on respondents’ SRH and socioeconomic characteristics in the 2011 wave, including age, gender, marital status, education level, economic status, pre-retirement occupation, and region.

Fixed effect models were used to examine the short-term effect of AHC on SRH over time. We used unweighted variables for the statistical analyses due to the following reasons. Firstly, the sampling weight in the CLHLS database is cross-sectional and varies over time for each individual. In fixed-effects models, sampling weights must be time-constant, so we could not incorporate the weights into the analysis [37]. Secondly, as the weight is only estimated based on elderly populations by age, gender and urban or rural residence, it is unable to reflect the national population distributions with respect to variables other than such characteristics [38]. Moreover, a weighted regression increases the standard errors in the analyses, making results potentially biased [30]. Two fixed-effect models were employed. Model 1 was a crude analysis without controlling for any confounding factors. Model 2 adjusted for socioeconomic characteristics, health status, health services access and health behaviors.

In the process of long-term effect detection, we extracted the lagged term of AHC in the 2011 wave and linked it with other variables in the 2018 wave, to avoid endogeneity (e.g., omitted explanatory variable, reverse causality). Two logistic regression models were employed. Model 1 was a crude analysis and Model 2 adjusted for multiple confounding variables.

Descriptive statistics were used for all study variables. A Chi-square test for categorical variables and a univariate ANOVA for continuous variables were used to compare respondents who did and did not attend AHC in unmatched and matched cohort. Matched weights were considered in all analyses in the matched groups. All analyses were performed using Stata software (version 15.0; StataCorp). All statistical tests were two-sided, with a significance threshold of 0.05.

## Results

Table 1 reports the participants’ characteristics broken down into waves in CLHLS. Of the 15,620 individuals, nearly half were males. Rural residents were more likely to be separated or divorced or widowed or never married, illiterate, and non-rich, with a lower prevalence of multiple chronic diseases, lower access to adequate healthcare services, and a lower rate of exercise, but higher rates of smoking and drinking, compared to urban residents. During the period 2011–2018, access to healthcare services among rural residents increased more than urban peers.

**TABLE 1:** Characteristics of participants by waves (N = 15,620) (Chinese Longitudinal Health Longevity Survey, China, 2011, 2014, 2018).

Variables	2011 wave		2014 wave		2018 wave	
	Rural	Urban	Rural	Urban	Rural	Urban
	(%/SD)	(%/SD)	(%/SD)	(%/SD)	(%/SD)	(%/SD)
N	4,221	3,907	2,556	2,493	1,172	1,271
Male	1,961	1,865	1,245	1,221	566	633
	(46.46)	(47.73)	(48.71)	(48.98)	(48.29)	(49.80)
Age, years	85.02	84.84	84.19	84.11	84.50	84.02
	(11.23)	(10.68)	(10.17)	(9.60)	(8.42)	(7.64)
Age≥80, years	2,727	2,494	1,578	1,526	802	844
	(64.61)	(63.83)	(61.74)	(61.21)	(68.43)	(66.40)
Married	1,605	1,522	1,063	1,050	482	558
	(38.2)	(39.07)	(42.07)	(42.48)	(41.52)	(44.36)
Illiterate	2,572	1,957	1,443	1,152	600	546
	(61.33)	(50.21)	(56.63)	(46.34)	(51.41)	(43.03)
White collar	301	1,265	196	788	84	351
	(8.28)	(33.88)	(9.04)	(32.89)	(8.73)	(29.62)
Region						
Eastern	1,581	1,470	920	1,031	453	464
	(37.46)	(37.62)	(35.99)	(41.36)	(38.65)	(36.51)
Central south	2,099	1,204	1,326	699	589	466
	(49.73)	(30.82)	(51.88)	(28.04)	(50.26)	(36.66)
West	327	671	182	436	74	226
	(7.75)	(17.17)	(7.12)	(17.49)	(6.31)	(17.78)
North	72	222	37	144	26	54
	(1.71)	(5.68)	(1.45)	(5.78)	(2.22)	(4.25)
Northeast	142	340	91	183	30	61
	(3.36)	(8.70)	(3.56)	(7.34)	(2.56)	(4.80)
Economic status						
Poor	682	556	334	253	147	98
	(16.28)	(14.32)	(13.18)	(10.20)	(12.66)	(7.78)
Neutral	2,871	2,584	1,865	1,711	784	867
	(68.52)	(66.56)	(73.6)	(68.99)	(67.53)	(68.81)
Richer	637	742	335	516	230	295
	(15.20)	(19.11)	(13.22)	(20.81)	(19.81)	(23.41)
ADLs disability	958	1,032	563	626	219	289
	(22.70)	(26.41)	(22.03)	(25.11)	(18.69)	(22.74)
IADLs disability	2,715	2,419	1,531	1,534	745	809
	(64.32)	(61.91)	(59.9)	(61.53)	(63.57)	(63.65)
Having social security and insurance	3,911	3,577	2,445	2,369	1,064	1,146
	(94.01)	(92.88)	(97.26)	(96.26)	(92.28)	(92.64)
Number of chronic diseases						
None	1,738	1,304	1,005	757	418	358
	(41.18)	(33.38)	(39.32)	(30.37)	(35.67)	(28.17)
One	1,127	982	692	652	352	400
	(26.70)	(25.13)	(27.07)	(26.15)	(30.03)	(31.47)
Two or more	1,356	1,621	859	1,084	402	513
	(32.13)	(41.49)	(33.61)	(43.48)	(34.30)	(40.36)
Access to healthcare service	3,914	3,710	2,424	2,400	1,116	1,230
	(93.06)	(95.57)	(95.51)	(97.05)	(96.46)	(98.32)
Cohabiting with others	3,327	3,185	1,944	2,030	865	980
	(79.42)	(81.92)	(76.96)	(81.56)	(76.68)	(80.59)
Smoking	855	691	475	461	201	198
	(20.34)	(17.75)	(18.66)	(18.58)	(17.27)	(15.78)
Drinking	781	638	431	388	181	192
	(18.63)	(16.52)	(17.02)	(15.66)	(15.7)	(15.45)
Exercising	1,120	1,717	551	1,003	303	481
	(26.89)	(44.32)	(21.96)	(41.38)	(26.23)	(38.73)

Note: SD or % is presented in parentheses.

ADLs, Activities of daily living; IADLs, Instrumental activities of daily living.

Among all respondents, AHC attendance increased from 31.96% in 2011 to 71.35% in 2018. Supplementary Figure S1 presents self-rated good health and AHC attendance in the different waves. The percentages of both increased from 2011 to 2018. Compared to their urban peers, rural old adults reported a lower rate of self-rated good health (81.81% vs. 82.90%, 83.26% vs. 83.59% and 85.32% vs. 86.39%, respectively in 2011, 2014 and 2018), but a higher rate of AHC attendance, except for the 2011 wave (30.92% vs. 33.09%, 62.25% vs.56.20% and 72.87% vs. 69.94%, respectively in 2011, 2014 and 2018). The growth rate of AHC attendance was 1.36% and 1.11% in the period 2011–2018, respectively for rural residents and urban residents. In addition, AHC was positively correlated with SRH (Cramer’s V was 0.053, 0.063 and 0.076, respectively, in 2011, 2014 and 2018; *p* < 0.001 in all waves).

Supplementary Table S1 presents the association between AHC and SRH among the elderly, before matching. As is shown in Model 1, AHC was significantly positively associated with SRH among rural respondents (OR = 1.395, *p* < 0.05). After controlling for potential confounders, the association was no longer significant among either rural or urban residents.

To examine the short-term effect of AHC on SRH, CEM was first used to balance the covariates of AHC attendance and non-attendance in the 2011 wave. The multivariate imbalance measure of L_1_ before and after CEM is reported in Supplementary Table S2. After matching, L_1_ was reduced from 0.424 to 1.151e-15, and all matched variables after CEM were also close to zero. It indicated a good matching performance.

Covariate imbalances in pre-matching and post-matching samples based on CEM in the baseline are presented in Table 2. The pre-matching group consisted of 3,435 rural residents and 2,095 urban residents, and most characteristics were significantly different between the two groups. After the CEM processing, 2,956 rural residents and 1,924 urban residents were retained, and most of characteristics were no longer significantly different.

**TABLE 2:** Covariate imbalance in pre-matching and post-matching samples based on coarsened exact matching (Chinese Longitudinal Health Longevity Survey, China, 2011).

Variables	Pre-matching (N = 5,530)			Post-matching (N = 4,880)		
	AHC		*p*-value	AHC		*p*-value^a^
	No	Yes		No	Yes	
	(%)	(%)		(%)	(%)	
N	3,435	2,095		2,956	1,924	
Self-rated health^b^			<0.001			0.987
Bad	740	313		403	262	
	(21.54)	(14.94)		(13.62)	(13.62)	
Good	2,695	1,782		2,553	1,662	
	(78.46)	(85.06)		(86.38)	(86.38)	
Gender^b^			<0.001			0.999
Male	1,481	1,013		1,438	936	
	(43.11)	(48.35)		(48.65)	(48.65)	
Female	1,954	1,082		1,518	988	
	(56.89)	(51.65)		(51.35)	(51.35)	
Age group^b^, years			<0.001			0.994
<80	714	1,130		1,529	995	
	(20.79)	(53.94)		(51.72)	(51.72)	
≥80	2,721	965		1,427	929	
	(79.21)	(46.06)		(48.28)	(48.28)	
Marital status^b^						0.998
Married	2,448	1,074		1,523	991	
	(71.54)	(51.46)		(51.59)	(51.59)	
Unmarried	974	1,013		1,429	930	
	(28.46)	(48.54)		(48.41)	(48.41)	
Education level^b^			<0.001			0.995
Illiterate	2,060	1,092		1,560	1,015	
	(60.25)	(52.32)		(52.84)	(52.84)	
Literate	1,359	995		1,392	906	
	(39.75)	(47.68)		(47.16)	(47.16)	
Pre-retirement occupation^b^			<0.001			0.990
Others	2,468	1,498		2,262	1,472	
	(78.72)	(83.92)		(85.61)	(85.61)	
White collar	667	287		380	247	
	(21.28)	(16.08)		(14.39)	(14.39)	
Region^b^			<0.001			1.000
Eastern	1,026	653		936	609	
	(29.87)	(31.17)		(31.65)	(31.65)	
Central south	1,525	1,068		1,552	1,010	
	(44.40)	(50.98)		(52.49)	(52.49)	
West	437	234		304	198	
	(12.72)	(11.17)		(10.29)	(10.29)	
North	147	60		63	41	
	(4.28)	(2.86)		(2.13)	(2.13)	
Northeast	300	80		101	66	
	(8.73)	(3.82)		(3.43)	(3.43)	
Economic status^b^			0.091			1.000
Poor	621	333		441	287	
	(18.22)	(15.93)		(14.92)	(14.92)	
Median	2,281	1,435		2,121	1,381	
	(66.93)	(68.63)		(71.81)	(71.81)	
Richer	506	323		392	255	
	(14.85)	(15.45)		(13.26)	(13.26)	
ADLs disability^b^			<0.001			<0.001
No	2,249	1,880		2,316	1,735	
	(66.72)	(91.8)		(79.98)	(91.88)	
Yes	1,122	168		580	153	
	(33.28)	(8.20)		(20.02)	(8.12)	
IADLs disability^b^			<0.001			<0.001
No	814	1,114		1,209	1,017	
	(23.72)	(53.25)		(41.00)	(52.83)	
Yes	2,617	978		1,741	908	
	(76.28)	(46.75)		(59.00)	(47.17)	
Having social security and insurance^b^			<0.001			0.029
No	316	113		201	102	
	(9.38)	(5.45)		(6.91)	(5.36)	
Yes	3,052	1,960		2,702	1,802	
	(90.62)	(94.55)		(93.09)	(94.64)	
Number of chronic diseases^b^			0.040			0.022
None	1,429	820		1,282	772	
	(41.60)	(39.14)		(43.36)	(40.12)	
One	857	585		735	541	
	(24.95)	(27.92)		(24.87)	(28.12)	
Two or more	1,149	690		939	611	
	(33.45)	(32.94)		(31.78)	(31.76)	
Access to healthcare services^b^			0.015			0.754
No	260	123		191	120	
	(7.62)	(5.90)		(6.51)	(6.27)	
Yes	3,154	1962		2,748	1793	
	(92.38)	(94.1)		(93.49)	(93.73)	
Cohabiting with others^b^			0.023			0.003
No	630	433		507	394	
	(18.4)	(20.91)		(17.25)	(20.67)	
Yes	2,793	1,638		2,432	1,513	
	(81.6)	(79.09)		(82.75)	(79.33)	
Smoking^b^			0.001			0.314
No	2,834	1,650		2,294	1,515	
	(82.84)	(79.10)		(77.90)	(79.12)	
Yes	587	436		651	400	
	(17.16)	(20.9)		(22.10)	(20.88)	
Drinking^b^			<0.001			0.223
No	2,884	1,687		2,422	1,554	
	(84.77)	(81.14)		(82.77)	(81.39)	
Yes	518	392		504	355	
	(15.23)	(18.86)		(17.23)	(18.61)	
Exercising^b^			0.003			0.513
No	2,365	1,357		1,968	1,268	
	(69.42)	(65.56)		(67.27)	(66.35)	
Yes	1,042	713		958	643	
	(30.58)	(34.44)		(32.73)	(33.65)	

a Considering match weights.

b Chi-square test.

Socioeconomic characteristics and SRH were pre-controlled using coarsened exact matching (in bold). To avoid the overmatching problem (e.g., a loss of statistical efficiency, loss of information, reduction of the power of the study), not all variables were included in the matching procedure. Socioeconomic characteristics and SRH variables were chose to match mainly because: (1) socioeconomic characteristics and health status are evidenced determinants of health check-up attendance; (2) socioeconomic characteristics are also the main factors influencing the ADLs, IADLs, medical insurance, chronic diseases, access to healthcare services and health behaviors; (3) SRH is a relatively comprehensive measure of health status.

Attending AHC or not was measured according to follow-up years (2014 and 2018 waves).

Note: AHC, Annual health check-ups; SRH, Self-rated health; ADLs, Activities of daily living; IADLs, Instrumental activities of daily living.

As is shown in Table 3, a total of 9,299 individuals comprised the matched cohort during the eight-year follow-up. The coverage rate of social security and insurance was higher among rural residents. Rural residents were more likely to be illiterate and the poor, with a lower prevalence of multiple chronic diseases, lower access to adequate healthcare services, and a lower rate of exercise, but higher rates of smoking and drinking, compared to urban residents.

**TABLE 3:** Characteristics of participants by waves in matched cohort (N = 9,299) (Chinese Longitudinal Health Longevity Survey, China, 2011, 2014, 2018).

Variables	2011 wave		2014 wave		2018 wave	
	Rural	Urban	Rural	Urban	Rural	Urban
	(%/SD)	(%/SD)	(%/SD)	(%/SD)	(%/SD)	(%/SD)
N	2,667	2,213	1,614	1,345	757	703
Male	1,179	998	764	642	366	348
	(44.21)	(45.10)	(47.34)	(47.73)	(48.35)	(49.50)
Age, years	85.57	85.84	84.47	84.92	84.75	84.26
	(11.33)	(10.72)	(10.27)	(9.96)	(8.57)	(8.14)
Age≥80, years	1,785	1,509	1,016	863	528	451
	(66.93)	(68.19)	(62.95)	(64.16)	(69.75)	(64.15)
Married	978	776	669	533	303	295
	(36.74)	(35.10)	(41.94)	(39.98)	(40.45)	(42.45)
Illiterate	1,656	1,202	914	659	384	313
	(62.23)	(54.32)	(56.77)	(49.00)	(50.93)	(44.52)
White collar	144	542	91	341	45	145
	(6.36)	(25.82)	(6.84)	(26.50)	(7.41)	(22.76)
Region						
Eastern	800	746	445	476	250	180
	(30.00)	(33.71)	(27.57)	(35.39)	(33.03)	(25.60)
Central south	1,582	850	1,008	486	444	337
	(59.32)	(38.41)	(62.45)	(36.13)	(58.65)	(47.94)
West	188	394	92	239	35	132
	(7.05)	(17.80)	(5.70)	(17.77)	(4.62)	(18.78)
North	27	94	20	60	11	20
	(1.01)	(4.25)	(1.24)	(4.46)	(1.45)	(2.84)
Northeast	70	129	49	84	17	34
	(2.62)	(5.83)	(3.04)	(6.25)	(2.25)	(4.84)
Economic status						
Poor	454	324	221	156	107	57
	(17.04)	(14.65)	(13.82)	(11.66)	(14.19)	(8.17)
Neutral	1,880	1,561	1,204	928	514	493
	(70.54)	(70.60)	(75.30)	(69.36)	(68.17)	(70.63)
Richer	331	326	174	254	133	148
	(12.42)	(14.74)	(10.88)	(18.98)	(17.64)	(21.20)
ADLs disability	578	567	363	334	138	160
	(21.67)	(25.62)	(22.49)	(24.83)	(18.23)	(22.76)
IADLs disability	1,727	1,417	951	842	477	439
	(64.75)	(64.03)	(58.92)	(62.60)	(63.01)	(62.45)
Having social security and insurance	2,448	1,992	1,550	1,266	692	628
	(93.08)	(91.50)	(97.42)	(95.55)	(92.14)	(91.81)
Number of chronic diseases						
None	1,204	854	701	460	298	209
	(45.14)	(38.59)	(43.43)	(34.20)	(39.37)	(29.73)
One	705	586	437	360	221	217
	(26.43)	(26.48)	(27.08)	(26.77)	(29.19)	(30.87)
Two or more	758	773	476	525	238	277
	(28.42)	(34.93)	(29.49)	(39.03)	(31.44)	(39.40)
Access to healthcare services	2,440	2,070	1,516	1,287	720	677
	(91.90)	(94.18)	(94.69)	(96.40)	(95.87)	(97.97)
Cohabiting with others	2,106	1,801	1,222	1,099	550	540
	(79.65)	(81.79)	(76.81)	(81.89)	(75.76)	(80.48)
Smoking	513	393	296	253	127	114
	(19.34)	(17.82)	(18.44)	(18.88)	(16.89)	(16.45)
Drinking	448	359	269	199	120	103
	(16.92)	(16.41)	(16.82)	(14.88)	(16.13)	(14.91)
Exercising	641	876	310	490	181	243
	(24.29)	(39.87)	(19.56)	(37.43)	(24.30)	(35.37)

Note: SD or % is presented in parentheses.

AHC, Annual health check-ups; ADLs, Activities of daily living; IADLs, Instrumental activities of daily living.

Table 4 presents the short-term effect of AHC on SRH among the elderly, after matching. As is presented in Model 1 and 2, old adults with AHC attendance predicted significantly higher SRH among rural respondents (OR = 1.947, *p* < 0.001 in Model 1; OR = 1.567, *p* < 0.05 in Model 2), whereas no significant results were detected among urban respondents (OR = 1.341, *p* > 0.05 in Model 1; OR = 1.602, *p* > 0.05 in Model 2).

**TABLE 4:** The effect of health check-ups on health among the elderly after coarsened exact matching (N = 9,299) (Chinese Longitudinal Health Longevity Survey, China, 2011, 2014, 2018).

Variables	Model 1		Model 2	
	Rural	Urban	Rural	Urban
	OR	OR	OR	OR
	(SE)	(SE)	(SE)	(SE)
AHC (ref: No)				
Yes	1.947***	1.341	1.567*	1.602^†^
	(0.361)	(0.302)	(0.343)	(0.457)
Age group, years (ref: <80)				
≥80			1.153	0.585
			(0.343)	(0.253)
Marital status (ref: unmarried)				
Married			0.792	0.590
			(0.283)	(0.291)
Economic status (ref: Poor)				
Median			2.497***	2.777**
			(0.527)	(1.027)
Richer			2.437*	3.551**
			(0.906)	(1.628)
ADLs disability (ref: No)				
Yes			0.621*	0.473*
			(0.146)	(0.145)
IADLs disability (ref: No)				
Yes			0.543**	0.433**
			(0.115)	(0.133)
Having social security and insurance (ref: No)				
Yes			1.224	0.698
			(0.474)	(0.382)
Number of chronic diseases (ref: None)				
One			0.835	0.723
			(0.174)	(0.234)
Two or more			0.633*	0.401**
			(0.139)	(0.132)
Access to healthcare services (ref: No)				
Yes			2.632**	2.986*
			(0.954)	(1.605)
Cohabiting with others (ref: No)				
Yes			1.055	0.790
			(0.285)	(0.381)
Smoking (ref: No)				
Yes			0.898	1.031
			(0.329)	(0.663)
Drinking (ref: No)				
Yes			0.741	1.133
			(0.284)	(0.528)
Exercising (ref: No)				
Yes			1.018	1.414
			(0.222)	(0.372)
Wave (ref: 2011 Wave)				
2014 Wave	0.464***	0.419***	0.593**	0.538*
	(0.071)	(0.078)	(0.115)	(0.132)
2018 Wave	0.394***	0.393***	0.440**	0.822
	(0.075)	(0.095)	(0.123)	(0.291)

Note: Model 1: Crude logistic regression; Model 2: Adjusted logistic regression.

Standard error presents in parentheses.

***p <0.001, **p <0.01, *p <0.05, ^†^ *p* < 0.1.

AHC, Annual health check-up; ADLs, Activities of daily living; IADLs, Instrumental activities of daily living.

Table 5 shows the long-term effect of AHC on SRH among the elderly. As is presented, the rural elderly who attended AHC in 2011 reported higher SRH in 2018 (OR = 2.044, *p* < 0.001 in Model 1; OR = 3.385, *p* < 0.001 in Model 2). However, the effect of AHC on SRH among the urban elderly was not significant (OR = 1.245, *p* > 0.1 in Model 1; OR = 1.628, *p* > 0.1 in Model 2).

**TABLE 5:** The effect of lagged health check-ups on health among the elderly (N = 2,544) (Chinese Longitudinal Health Longevity Survey, China, 2011, 2018).

Variables	Model 1		Model 2	
	Rural	Urban	Rural	Urban
	OR	OR	OR	OR
	(SE)	(SE)	(SE)	(SE)
AHC in 2011 (ref: No)				
Yes	2.044***	1.245	3.385***	1.628
	(0.411)	(0.212)	(1.213)	(0.524)
Gender (ref: Male)				
Female			1.081	1.503
			(0.360)	(0.495)
Age group, years (ref: <80)				
≥80			2.699**	1.283
			(0.974)	(0.453)
Marital status (ref: unmarried)				
Married			1.763	0.754
			(0.630)	(0.275)
Education level (ref: illiterate)				
Literate			0.611	0.912
			(0.215)	(0.321)
Pre-retirement occupation (ref: white collar)				
Others			1.248	1.531
			(0.605)	(0.554)
Region (ref: Eastern)				
Central south			1.548	0.657
			(0.555)	(0.231)
West			0.727	0.720
			(0.386)	(0.314)
North			1.727	1.091
			(1.974)	(0.847)
Northeast			4.075	1.783
			(3.513)	(1.299)
Economic status (ref: Poor)				
Median			4.044***	3.608*
			(1.554)	(1.803)
Richer			4.303**	7.216**
			(2.166)	(4.480)
ADLs disability (ref: No)				
Yes			0.266***	0.430*
			(0.102)	(0.148)
IADLs disability (ref: No)				
Yes			0.823	0.335**
			(0.308)	(0.142)
Having social security and insurance (ref: No)				
Yes			1.241	1.410
			(0.671)	(0.773)
Number of chronic diseases (ref: None)				
One			0.722	0.300*
			(0.292)	(0.146)
Two or more			0.409*	0.236**
			(0.151)	(0.110)
Access to healthcare services (ref: No)				
Yes			1.751	5.445^†^
			(1.120)	(4.737)
Cohabiting with others (ref: No)				
Yes			0.853	1.727
			(0.341)	(0.683)
Smoking (ref: No)				
Yes			1.345	0.713
			(0.651)	(0.335)
Drinking (ref: No)				
Yes			1.348	2.986^†^
			(0.670)	(1.774)
Exercising (ref: No)				
Yes			1.570	1.127
			(0.597)	(0.400)

Note: AHC was extracted from 2011 wave and SRH was extracted from 2018 wave.

Model 1: Crude logistic regression; Model 2: Adjusted logistic regression.

Standard error presents in parentheses.

***p <0.001, **p <0.01, *p <0.05, ^†^ *p* < 0.1.

AHC, Annual health check-up; ADLs, Activities of daily living; IADLs, Instrumental activities of daily living.

## Discussion

To our knowledge, this is the first study using a nationally representative sample with longitudinal data to examine the effect of health check-ups on health among elderly Chinese, and the urban–rural differences. The CEM approach to preprocess the data enabled the reduction of bias caused by large socioeconomic and health status disparities that influence residents’ preventive care utilization [1, 18, 31]. The lagged explanatory variable avoided the endogeneity [39]. We found that: 1) AHC attendance among old adults increased during the eight-year follow-up; 2) utilization of AHC and the growth rate of AHC attendance were higher among rural residents; 3) AHC only had a positive effect on SRH in the rural elderly; 4) the magnitude and the significance of the effect became larger in the long-term effect detection.

Among all respondents, AHC utilization increased from 31.96% in 2011 to 71.35% in 2018. Consistent with our finding, a previous study using CLHLS data conducted in 2011 showed that 34.72% of elderly people underwent AHC [40], and it increased to 67% in 2017 [41]. Following China’s rapid economic growth and social development, with its accompanying enhanced health beliefs, per capita subsidies for BPHS were increased from CNY 15 in 2009 to CNY 55 in 2018 (USD 1 = CNY 6.50). It implies government endeavors in achieving the universal access to public health. From the perspective of residents, free AHC provision is one of the most effective and affordable approaches for them to meet essential healthcare needs, with no limitation due to socioeconomic condition and no worries about cost.

It is worth noting that AHC utilization and the growth rate of AHC attendance were higher among rural old adults. It might be considered the manifestation of a huge health need release, previously hidden in the billed services model. Compared to their urban peers, the rural elderly are likely to have lower educational attainment and household income [16, 42], as well as insufficient financial support [43]. Free AHC increases the low-income group’s accessibility to preventive care.

As an elder care project, AHC is one health promotional strategy for a healthy ageing, contributing to enhanced health, life satisfaction, and quality of life [44]. In this study we found that rural old adults who participated in AHC were more likely to report good SRH than those who did not, in line with previous studies [44, 45]. The larger and more significant results of the effect of the lagged AHC on SRH among the rural elderly further indicated that the benefit of health check-ups to health promotion may be getting greater over time. Routine health check-ups help the elderly to understand their recent overall health and exposure to potential diseases. The guidelines on health maintenance and disease control from healthcare staff increase the likelihood of better health in their later life. As improved health is related to decreased curative demand, utilization and expenditure of outpatient and inpatient service might also be reduced [46]. It needs to be further addressed in the future.

The findings that the positive effect of AHC on SRH was significant only among rural respondents, and the urban-rural differences became larger when using the lagged AHC may be attributed to the increase in access to preventive care services for the rural elderly and the compliance disparities between rural and urban old adults. On the one hand, cost is regarded as a considerable barrier to the uptake of healthcare services. The rural old adults tend to have less stable and disposable income. They would forsake much needed preventive care and medical care services, when they have to sacrifice their basic living budget (e.g., food, housing consumes, transportation) for these services [18]. AHC under NBPHS program helps the poor and the senior, increasing their opportunities to make use of preventive care services, without the cost worry [47]. It helps find and intervene risk factors in early stage. On the other hand, better compliance is good for health promotion. Non-compliance with medication occurs frequently in the treatment of chronic conditions [48], and it’s easier happened among the urban population [49, 50]. Further, good compliance was found in individuals with a single illness, compared to multiple illnesses [51]. As the findings indicated a higher prevalence of two or more chronic diseases among the urban elderly, they are more likely to face complex treatments regimes. It has been demonstrated that treatments of long duration therapies, various medicines with complex dosing regimes, adverse effects caused by long-term use, and preoccupation with study or work are the key reasons for lack of compliance [51, 52]. Thus, to some extent, better compliance for rural elderly people may partly explain the observation of a significant effect of health check-ups on health and the urban-rural differences. However, the exact mechanism of the positive relationship between health check-ups and health for old adults in rural area needs to be further examined in future studies.

### Limitations

We acknowledge several limitations in this study. Firstly, this study relied on SRH to measure health, which cannot reflect comprehensive health conditions, although it has proven to be a good indicator of objective health, particularly for the elderly [21, 53]. It may also lead to potential recall bias in responses. Secondly, although we demonstrated that the positive effect of health check-ups on health was obvious by employing matching method and lagged explanatory variable, further research is needed to identify the rigorous causal effect of health check-ups on health. Thirdly, this study was unable to examine the possible causes and mechanisms of urban–rural differences. Resident’s awareness and initiative, compliance with prescribed treatment regimes, the strength of the government’s policy implementation and publicity campaigns, and the quality of preventive care facilities are all associated with health check-ups utilization and effect on health status. The determinants of urban–rural differences in the relationship between health check-ups and health need to be examined in further studies. Despite these limitations, our study may still be relevant for the provision of important evidence on the relationship between health check-ups and health among old adults, and the urban-rural differences in China.

### Conclusion

The present study highlights the increase in AHC utilization among the elderly in China, and a higher utilization of AHC in rural area, as well as the positive effect of AHC on SRH among rural old adults. Our findings indicate enhanced access to public healthcare services and health check-ups’ essential role in health promotion on the background of the NBPHS. The increasing AHC utilization is beneficial in progressively equalizing BPHS, and in gradually reducing urban–rural gaps in access to healthcare services as well as income-related health inequalities [54]. We would recommend that policies aim at strengthening publicity about free health check-ups and improving residents’ awareness of preventive care, particularly among old adults. The government should further increase public health investment and make more efforts toward enhancing universal health.

## Abbreviations

ADLs, activities of daily living; AHC, annual health check-up; BMI, body mass index; BP, blood pressure; BPHS, basic public health services; CEM, coarsened exact matching; CLHLS, Chinese Longitudinal Healthy Longevity Survey; IADLs, instrumental activities of daily living; NBPHS, national basic public health service program; NCDs, non-communicable diseases; PSM, propensity score matching SRH, self-rated health; USPSTF, The U.S. Preventive Services Task Force; WHO, World Health Organization.
