# Supplementary material for: The Effect of Health Check-Ups on Health Among the Elderly in China: Evidence From 2011–2018 Longitudinal Data
Source: Int J Public Health. 2022 Aug 5;67:1604597. doi: 10.3389/ijph.2022.1604597 (PMC9389946; doi:10.3389/ijph.2022.1604597)
Supplement: Supplementary file 1 [file Table1.docx]

**International Journal of Public Health**

**The effect of health check-ups on health among the elderly in China: Evidence from 2011-2018 longitudinal data**

Table S1 The association between health check-ups and health among the elderly before coarsened exact matching (N = 15,620). Chinese Longitudinal Health Longevity Survey, China, 2011, 2014, 2018.

| Variables | Model 1 | |  | Model 2 | |
| --- | --- | --- | --- | --- | --- |
|  | Rural | Urban |  | Rural | Urban |
|  | OR | OR |  | OR | OR |
|  | (SE) | (SE) |  | (SE) | (SE) |
| AHC (ref: No) |  |  |  |  |  |
| Yes | 1.395^*^ | 1.078 |  | 1.239 | 1.156 |
|  | (0.186) | (0.149) |  | (0.187) | (0.193) |
| Age group, years (ref: <80) |  |  |  |  |  |
| ≥80 |  |  |  | 1.874^*^ | 0.987 |
|  |  |  |  | (0.502) | (0.282) |
| Marital status (ref: unmarried) |  |  |  |  |  |
| Married |  |  |  | 0.922 | 0.594 |
|  |  |  |  | (0.275) | (0.205) |
| Economic status (ref: Poor) |  |  |  |  |  |
| Median |  |  |  | 2.044^***^ | 2.007^**^ |
|  |  |  |  | (0.382) | (0.470) |
| Richer |  |  |  | 1.953^*^ | 2.605^**^ |
|  |  |  |  | (0.558) | (0.781) |
| ADLs disability (ref: No) |  |  |  |  |  |
| Yes |  |  |  | 0.668^*^ | 0.631^*^ |
|  |  |  |  | (0.120) | (0.130) |
| IADLs disability (ref: No) |  |  |  |  |  |
| Yes |  |  |  | 0.596^**^ | 0.437^***^ |
|  |  |  |  | (0.104) | (0.0873) |
| Having social security and insurance (ref: No) |  |  |  |  |  |
| Yes |  |  |  | 1.038 | 1.708 |
|  |  |  |  | (0.320) | (0.565) |
| Number of chronic diseases (ref: None) |  |  |  |  |  |
| One |  |  |  | 0.852 | 0.695 |
|  |  |  |  | (0.151) | (0.160) |
| Two or more |  |  |  | 0.618^**^ | 0.380^***^ |
|  |  |  |  | (0.110) | (0.0868) |
| Access to healthcare services (ref: No) |  |  |  |  |  |
| Yes |  |  |  | 1.994^*^ | 2.732^*^ |
|  |  |  |  | (0.596) | (1.170) |
| Cohabiting with others (ref: No) |  |  |  |  |  |
| Yes |  |  |  | 0.918 | 1.285 |
|  |  |  |  | (0.193) | (0.413) |
| Smoking (ref: No) |  |  |  |  |  |
| Yes |  |  |  | 0.977 | 1.207 |
|  |  |  |  | (0.270) | (0.443) |
| Drinking (ref: No) |  |  |  |  |  |
| Yes |  |  |  | 1.656^†^ | 1.397 |
|  |  |  |  | (0.486) | (0.448) |
| Exercising (ref: No) |  |  |  |  |  |
| Yes |  |  |  | 1.210 | 1.304 |
|  |  |  |  | (0.214) | (0.228) |
| Wave (ref: 2011 Wave) |  |  |  |  |  |
| 2014 Wave | 0.839 | 0.747^**^ |  | 0.913 | 0.878 |
|  | (0.090) | (0.079) |  | (0.118) | (0.118) |
| 2018 Wave | 0.682^**^ | 0.758^†^ |  | 0.692^†^ | 1.285 |
|  | (0.098) | (0.113) |  | (0.132) | (0.285) |

Note: Model 1: Crude logistic regression; Model 2: Adjusted logistic regression.

AHC = Annual Health Check-up; ADLs = activities of daily living; IADLs = instrumental activities of daily living.

Standard error presents in parentheses

^***^ p<0.001, ^**^ p<0.01, ^*^ p<0.05, ^†^p<0.1
