# Supplementary material for: The Effect of Health Check-Ups on Health Among the Elderly in China: Evidence From 2011–2018 Longitudinal Data
Source: Int J Public Health. 2022 Aug 5;67:1604597. doi: 10.3389/ijph.2022.1604597 (PMC9389946; doi:10.3389/ijph.2022.1604597)
Supplement: Supplementary file 2 [file Table2.docx]

**International Journal of Public Health**

**The effect of health check-ups on health among the elderly in China: Evidence from 2011-2018 longitudinal data**

Table S2 The L_1_ statistic before and after coarsened exact matching. Chinese Longitudinal Health Longevity Survey, China, 2011.

| Variables | Before Matching (N=5530) | After Matching (N=4880) |
| --- | --- | --- |
|  | L_1_(mean) | L_1_(mean) |
| Gender | 0.051 (-0.051) | 8.0e-16 (3.1e-15) |
| Age group | 0.329 (-0.329) | 5.0e-16 (1.3e-14) |
| Marital status | 0.192 (0.192) | 4.2e-16 (6.1e-16) |
| Education level | 0.074 (0.074) | 1.7e-15 (-1.2e-15) |
| Pre-retirement occupation | 0.050 (0.050) | 2.5e-15 (4.3e-15) |
| Region | 0.071 (-0.212) | 2.5e-15 (1.3e-14) |
| Economic status | 0.022 (0.012) | 2.7e-15 (3.3e-15) |
| Self-rated health | 0.059 (0.059) | 6.9e-16 (1.0e-15) |
| Multivariate L_1_ | 0.424 | 1.151e-15 |

Note: The mean is labeled in parentheses and reports the difference in means.
