# Supplementary material for: The Effect of Health Check-Ups on Health Among the Elderly in China: Evidence From 2011–2018 Longitudinal Data
Source: Int J Public Health. 2022 Aug 5;67:1604597. doi: 10.3389/ijph.2022.1604597 (PMC9389946; doi:10.3389/ijph.2022.1604597)
Supplement: Supplementary file 3 [file Image1.pdf]

**International Journal of Public Health**

**The effect of health check-ups on health among the elderly in China: Evidence from 2011-2018 longitudinal data**

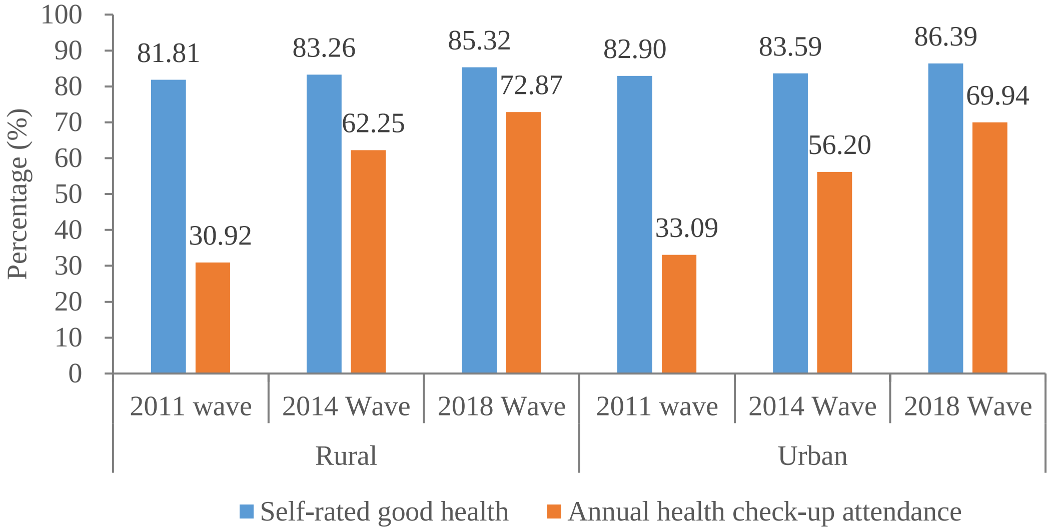


Figure S1 Self-rated good health and the utilization of annual health check-up by waves (N = 15,620). Chinese Longitudinal Health Longevity Survey, China, 2011, 2014, 2018.
